# Educational attainment and endometrial cancer: A Mendelian randomization study

**DOI:** 10.3389/fgene.2022.993731

**Published:** 2022-11-29

**Authors:** Qixia Wang, Runchen Wang, Chao Chen, Yi Feng, Zhiming Ye, Miaorong Zhan, Hao Wen, Kaimin Guo

**Affiliations:** ^1^ Department of Obstetrics and Gynecology, Guangzhou Women and Children’s Medical Center, Guangzhou Medical University, Guangzhou, China; ^2^ Nanshan School, Guangzhou Medical University, Guangzhou, China; ^3^ Third Clinical School, Guangzhou Medical University, Guangzhou, China; ^4^ First Clinical School, Guangzhou Medical University, Guangzhou, China

**Keywords:** educational attainment, Mendelian randomization (MR), endometrial cancer (EC), causal inference, risk factor

## Abstract

**Background:** Low educational attainment has been reported as a risk factor for many diseases. However, conclusion on the association between educational attainment and endometrial cancer (EC) are inconsistent in previous observational studies. This study aims to explore the potential causal association between educational attainment and EC.

**Methods:** A Mendelian Randomization analysis was performed using publicly summary-level data sets of genome-wide association studies (GWAS). A total of 306 single-nucleotide polymorphisms (SNPs) were extracted as instrumental variables for the exposure of educational attainment from the Social Science Genetic Association Consortium GWAS summary data of 1,131,881 participants of European ancestry. SNPs of EC were obtained from the Endometrial Cancer Association Consortium, the Epidemiology of Endometrial Cancer Consortium and the UK Biobank involving 121,885 people. We conducted inverse variance weighted (IVW) to estimate the causal effect as our primary outcome. And we perform several sensitivity analyses, including MR-Egger regression, weighted median method, MR-PRESSO (Mendelian Randomization Pleiotropy Residual Sum and Outlier) global test, and leave-one-out sensitivity analysis, to evaluate the effect of pleiotropism on the causal estimates.

**Results:** Genetic predisposition towards 4.2 years of additional educational attainment was associated with 38% lower risk of EC. (odds ratio 0.72, 95% confidence interval 0.62 to 0.83; *p* = 1.65*10^−5^). The consistent results of sensitivity analyses indicated our causal estimates were reliable. Genetic predisposition towards longer educational attainment was associated with lower risk of obesity, high waist-to-hip ratio (WHR), and diabetes.

**Conclusion:** This study indicated that low educational attainment was a causal risk factor for EC, especially for EC with endometrioid histology. Low educational attainment might lead to EC through the mediator of obesity, high WHR, and diabetes.

## Background

Endometrial cancer (EC) is one of the most common gynecological tumors. There are more than 417,000 new cases and approximately 98,000 deaths attributed to EC worldwide in 2020 (Cancer Trends, 2020). According to the International Agency for research on Cancer, the incidence rate of EC is increasing rapidly and is estimated to increase by more than 50% worldwide by 2040 (Morice et al., 2016a; World Health OrganiZation, 2020). It has been generally accepted that obesity, diabetes, early age at menarche, nulliparity, late-onset menopause, older age (≥55 years), and use of tamoxifen are common risk factors of EC (Morice et al., 2016b). However, substantial uncertainty still surrounds other potential factors, such as educational attainment. Although low educational attainment has been reported as causal risk factor for many diseases (Mountjoy et al., 2018; Zhou et al., 2019), the conclusions on the association between educational attainment and EC are inconsistent in previous observational studies (Wynder et al., 1966; Mouw et al., 2008). Besides, observational studies cannot definitively determine causality, and the conclusions of which may be subject to the potential limitations of observational study, including confounding, reverse causation, and measurement error (Davey Smith and Ebrahim, 2001; Boyko, 2013). Indeed, clarifying the causal association between educational attainment and EC has widespread implications for the understanding of the causes and preventive approaches of EC. Although Randomized controlled trials (RCTs) are the gold standard to establish causal relationships, it is not feasible for this study. It usually takes a long latency period before the causal effect between exposure (educational attainment) and outcome (EC) can be seen. Besides, it needs to limit education to some of the participants inevitably, which is unethical (Black, 1996). Therefore, innovative approaches are urgently needed to assess the causal inference.

Mendelian randomization (MR) study is an approach that can be applied to evaluate the causality between various phenotypes and disease outcomes by using genetic variant as instrumental variables (IVs) (Lawlor et al., 2008). These genetic variants are largely independent of confounders, as they are randomly allocated from conception (Emdin et al., 2017). Therefore, MR study may have a similar effect on evaluating causality, compared with RCTs (Katikireddi et al., 2018).

In this study, we were motivated to investigate whether educational attainment had a causal effect on EC by performing a Mendelian Randomization study.

## Materials methods

### Genetic variants associated with educational attainment

The definition of educational attainment is the number of years of schooling that individuals had completed, which was the same algorithm as the latest and largest educational attainment GWAS from Social Science Genetic Association Consortium (SSGAC) (Lee et al., 2018). We obtained SNPs of educational attainment from the SSGAC’s GWAS summary data (Lee et al., 2018), which was a meta-analysis of 71 discovery cohorts including 1,131,881 participants of European ancestry as shown in Supplementary Table S1. The IVs used in MR study must satisfy the following three assumptions: 1) It is closely related to the exposure; 2) It is not associated with confounders; 3) It only affects the outcome through the exposure pathway (Supplementary Figure S1). The SNPs will be chosen to be IVs after we clumping all SNPs that were associated with educational attainment at a genome-wide significance threshold (*p* < 5.0 × 10^−8^) and were not in linkage disequilibrium (*r* ^2^ < 0.001 and distance>10,000 kb) with other SNPs. After harmonization and removal of palindromic SNPs with intermediate allele frequencies, we selected 306 independent SNPs to be IVs associated with educational attainment (Supplementary Table S2). To evaluate whether the selected IVs were strong enough to explain educational attainment, we calculated F-statistic to evaluate the strength of the IVs (Supplementary Table S2). IVs with an F-statistic <10 were regarded as weak instruments (Burgess et al., 2017). F-statistic was calculated with the formula: F-statistic = *R* ^2^ × (N−2)/(1−*R* ^2^), where *R* ^2^ is the variance of the phenotype explained by each genetic variant in exposure. *R* ^2^ was calculated with the formula: *R* ^2^ = 2 × (Beta)2 × EAF × (1−EAF)/[2 × (Beta)2 × EAF × (1−EAF) + 2 × (SE)2 × N × EAF × (1−EAF)] (Shim et al., 2015). To evaluate whether the sample size of the selected was sufficient, we conducted Power calculations with an odds ratio of 0.72 educational attainment (Supplementary Table S3) (Brion et al., 2013).

### Genetic variants associated with endometrial cancer

We obtained the SNPs associated with EC from the endometrial GWAS data contributed by Endometrial Cancer Association Consortium (ECAC), the Epidemiology of Endometrial Cancer Consortium (E2C2) and the UK Biobank, which was a meta-analysis including 12,906 EC cases and 108,979 country-matched controls of European ancestry as shown in Supplementary Table S1 (O'Mara et al., 2018). In this meta-analysis, EC was further divided by histology type, including 8,758 cases of endometrial cancer with endometrioid histology (EEC) and 1,230 cases of endometrial cancer with non-endometrioid histology (NEC) (Supplementary Table S1). For each of the 306 SNPs associated with educational attainment, we retrieved summary data from the endometrial GWAS data mentioned above (Supplementary Table S2).

### Statistical analyses

To investigate whether educational attainment play a causal role in EC, we perform MR analyses. Firstly, we conducted a random effects inverse variance weighted (IVW) meta-analysis of the Wald ratio for individual SNPs. The Wald ratio was an estimate of causal effect of each IV, which was calculated as the ratio of Beta for SNP in outcome data divided by Beta for the same SNP in the exposure data. IVW method was the main approach to estimate the overall causal effect of the exposure on the outcome. The causal effects were calculated as the odds ratio (OR) for the risk of endometria cancer per one standard deviation (SD) increase in educational attainment (one SD is equivalent to 4.2 years).

Secondly, we conducted a series of sensitivity analyses, including MR-Egger regression, weighted median method, MR-PRESSO (Mendelian Randomization Pleiotropy Residual Sum and Outlier) global test, and leave-one-out sensitivity analysis, to evaluate the effect of pleiotropism on Mendelian random causality, relaxing the Mendelian randomization hypothesis partly. And we also measured the heterogeneity between the causal estimates across all SNPs in the IVW method by calculating the Cochran’s Q statistic to provides more reliability for a causal effect. The MR-Egger regression method was used to assess the horizontal pleiotropic effect of IVs. When horizontal pleiotropy occurs, and MR-Egger regression intercept significantly differs from zero (*p* < 0.05). In the case of violation of Mendelian randomization assumptions, the weighted median method was used to provide valid causal estimate. The weighted median method was able to provide consistent effect estimates even if up to 50% of genetic variants do not conform to the instrument strength independent of direct effect assumption (Bowden et al., 2016). The MR-PRESSO global test was used to investigate whether there was outlier of IV whose variant-specific causal estimates differ significantly from those of other selected IVs. Leave-one-out sensitivity analysis was conducted to assess the reliability of the causal estimates came from IVW method by removing each SNP from the analysis and re-estimating the causal effect.

Thirdly, to check whether the genetic risk of EC may also forecast the outcome of educational attainment, we conducted a reverse Mendelian randomization analysis using 17 SNPs associated with EC and 9 SNPs associated with EEC. Under conditions of massive pleiotropy, genetic risk of EC might also predict educational attainment outcomes.

Lastly, to investigate the potential mechanisms from educational attainment to EC, we conducted Mendelian randomization analysis to investigate whether genetic predisposition towards higher educational attainment could lead to improvements in the established EC risk factors. In this study, we investigated three common risk factors for EC, including obesity, waist-to-hip ratio (WHR), and diabetes, whose SNPs could be obtained in GWAS summary data (Supplementary Table S4).

We notice that some possible overlap between the exposure GWAS participants and the outcome GWAS participants as shown in Supplementary Table S1. Therefore, we used another set of 71 SNPs associated with educational attainment in the dataset of SSGAC with a population of 293,723 participants to perform all Mendelian randomization analyses on EC again (Supplementary Tables S1, S6) (Okbay et al., 2016).

All analyses were conducted in R (version 4.2.1).

## Results

### Causal effect from educational attainment to endometrial cancer

We retrieved 306 SNPs associated with educational attainment (Supplementary Table S2). The F-statistic of every IV was >10, suggesting that the no weak IV existed in our study (Supplementary Table S2). The number required for 80% power in endometrial with an OR of 0,72 is at least 74,894 subjects (Supplementary Table S3). The 306 SNPs selected in our study involve a population of 1,131,881 people, which is sufficient to derive unbiased causal estimates.

After performing Mendelian randomization analysis, we found that 1 SD longer educational attainment was associated with a 38% lower risk of EC [OR (odds ratio), 0.72; 95% CI, 0.62–0.83; *p* = 1.65 × 10^−5^] (Table 1). Weighted-median method (OR, 0.75; 95% CI, 0.60–0.93; *p* = 8.67 × 10^−3^) and MR-Egger regression (OR, 0.56; 95% CI, 0.31–1.03; *p* = 0.06) method also showed overall consistent protective effects for educational attainment on EC, although these two methods usually provided less precise estimates than with IVW method (Table 1). Individual causal estimates from each of the 306 SNPs in the above 3 MR methods were shown in Supplementary Figure S2.

**TABLE 1:** Causal effect from educational attainment to endometrial cancer.

Method	Number of SNPs	OR	95% CI	p-value	
Endometrial cancer					
IVW method	306	0.72	0.62–0.83	1.65 × 10−5	
Weighted-median method	306	0.75	0.60–0.93	0.008	
MR-PRESSO test	306	0.71	0.62–0.83	2.22 × 10−5	Global test *p* = 0.165
MR Egger regression	306	0.56	0.31–1.03	0.06	
Endometrial cancer with endometrioid histology					
IVW method	306	0.66	0.55–0.78	2.79 × 10−6	
Weighted-median method	306	0.68	0.53–0.89	<0.01	
MR-PRESSO test	306	0.72	0.55–0.78	4.21 × 10−6	Global test *p* = 0.238
MR Egger regression	306	0.37	0.18–0.74	<0.01	
Endometrial cancer with non-endometrioid histology					
IVW method	305	0.99	0.65–1.51	0.96	
Weighted-median method	305	1.09	0.60–2.01	0.77	
MR-PRESSO test	305	0.99	0.66–1.48	0.95	Global test *p* = 0.827
MR Egger regression	305	3.70	0.69–19.84	0.13	

IVW, inverse variance weighted; MR-PRESSO, MR, pleiotropy residual sum and outlier; OR, odds ratio; CI, confidence interval; SNPs, single nucleotide polymorphisms.

In sensitivity analyses, the intercept from the MR-Egger regression analysis was 0.003 (*p* = 0.418), which suggested that no apparent evidence of overall directional pleiotropy in our causal results (Table 1). The Cochrane’s Q statistic for our IVW method was 329.70 (*p* = 0.158), which suggested that the result of IVW method in our study, which is the main result of our study, could provide reliability for causal effect with low heterogeneity. The funnel plot of the selected 306 SNPs showed general symmetry, suggesting little evidence of heterogeneity or pleiotropy in our causal estimates (Supplementary Figure S3). The weighted median method indicated that more than half of the instrumental SNPs in our analysis satisfied the IV assumptions. MR-PRESSO global test (*p* = 0.165) and leave-one-out sensitivity analysis suggested the lack of an outlier SNP whose variant-specific causal estimate differed substantially from those of other SNPs (Table 1; Supplementary Figure S4).

In the further analyses of EC subtypes, we found that educational attainment was more causally associated with EEC (OR, 0.66; 95% CI, 0.55–0.78; *p* = 2.79 × 10^−6^), while educational attainment showed no causal association with NEC histology (OR, 0.99; 95% CI, 0.65–1.51; *p* = 0.96) (Table 1; Supplementary Figures S5, S6).

After finishing all the analyses mentioned above, we used another set of 71 SNPs that were associated with educational attainment in the dataset of SSGAC with a population of 293,723 participants to perform all Mendelian randomization analyses again to verify the reliability of our results (Supplementary Table S6). Finally, we obtained consistent results with the results mentioned above which indicated that our causal estimates were robust (Supplementary Table S5; Supplementary Figure S7–S9). Since the result come from these two datasets were consistent, we remain decided to use the larger set of instruments (with 306 SNPs) in our main analyses to maintain sufficient statistical power for our sensitivity analyses.

### Causal effect from endometrial cancer to educational attainment

In reverse Mendelian randomization study, we found little evidence for the hypothesis that EC may have the reverse effect on educational attainment, because 3 MR methods including IVW method, MR-Egger method, and Weighted Median method showed consistent result (Supplementary Table S7).

### Causal effect from educational attainment to endometrial risk factors

To identify the potential risk factors that could mediate the association between educational attainment and EC, we investigated whether educational attainment was associated with established EC risk factors. Table 2 showed that, in IVW method, 1 SD longer educational attainment was associated with 46% lower odds of obesity class 1, 51% lower odds of obesity class 2, 64% lower odds of obesity class 3, 54% lower odds of type 1 diabetes, 47% lower odds of type 2 diabetes, 0.23 lower WHR (Table 2).

**TABLE 2:** Causal effect from educational attainment to endometrial cancer risk factors.

Method	Number of SNPs	Causal effect	95% CI	*p*-value
Obesity				
Obesity class 1	246	0.54	0.45–0.64	2.69 × 10^−12^
Obesity class 2	246	0.49	0.38–0.62	1.03 × 10^−8^
Obesity class 3	246	0.36	0.23–0.55	2.66 × 10^−6^
Waist-to-hip ratio	247	−0.23	−0.29–0.17	2.09 × 10^−12^
Waist-to-hip ratio (adjusted for BMI)	248	−0.13	−0.17–0.08	5.35 × 10^−8^
Diabetes				
Type 1 diabetes	263	0.46	0.30–0.71	5.0 × 10^−4^
Type 2 diabetes	264	0.53	0.47–0.60	4.51 × 10^−26^
Type 2 diabetes (adjusted for BMI)	31	0.64	0.45–0.92	0.02

*SNPs, associated with educational attainment were obtained from SSGAC, of 2018.

*Causal effect for continuous risk factors were expressed as absolute values and as odds ratio for binary traits IVW, inverse variance weighted. MR-PRESSO, MR, pleiotropy residual sum and outlier; OR, dds ratio; CI, confidence interval; SNPs, single nucleotide polymorphisms; BMI, body mass idex.

## Discussion

In this Mendelian randomization study, we found strong genetic support that educational attainment had a robust causal relationship with EC, especially for EEC. More specifically, 4.2 years longer educational attainment can predict 38% reduction in the risk of EC.

Previous studies have tried to investigate the association between educational attainment and EC. Jensen and his colleagues found no difference in the incidence rate of EC with different level of educational attainment (Jensen et al., 2008). In contrast to our study, Mouw and his colleagues identified that women with lower educational attainment had a lower risk of EC (Mouw et al., 2008). Laaksonen and his colleagues found that the body fatness-related EC burden was high among women with lower educational attainment in Australia (Laaksonen et al., 2019). To date, there is no consistent conclusion on the association between educational attainment and EC. Most of the evidence mentioned above came from observational studies, which could not rule out residual confounding completely and derive causality. For example, confounding factors other than obesity were lack of control in the study of Mouw.

In fact, many intermediate phenotypes can mediate the association between educational attainment and EC, though the exact mechanisms mediating this association is elusive. In this study, we found that higher educational attainment reduced the risk of obesity, high WHR, and diabetes, which suggested that these three factors might be an intermediate factor between educational attainment and EC. Besides, educational attainment, as an important indicator of socioeconomic status, affects people’s life circumstances and health (Galobardes et al., 2007). Broadly consistent with our hypothesis and findings, previous study has proved that people with higher educational attainment have lower risk of obesity and diabetes than those with lower educational attainment (Lawrence, 2017). Another study found that reform to education policy in the UK have led to a reduction in the risk of body mass index and diabetes (Davies et al., 2018). Therefore, the interference of the potential confounding factors mentioned above may lead to the deviation of the results in previous observational studies. The accuracy of the previous conclusions needs to be further confirmed. However, whether the risk factors of EC are intermediate factor on the educational attainment-EC pathway needs further studies to explore-for example, by applying two step Mendelian randomization. Although the exact mechanism is still unknown, we should not ignore the importance of improving education to reduce EC risk, as our MR analysis has already provided a potential mechanism between educational attainment and endometrial cancer.

There are two histological subtypes of EC, including EEC and NEC. NECs include serous carcinoma, mucinous carcinoma, clear cell carcinoma and carcinosarcoma. Therefore, to investigate the relationship between educational attainment and EC, it is necessary to analyze subtypes of EC separately. Interestingly, significant causal relationship was found in overall EC group and EEC group, while no causal effect was seen in the NECs group. This might be attributed to the different clinical and pathological features between EEC and NECs. EEC usually occurs in younger women, often obese or diabetic, while NEC mainly occurs in elderly women who are neither obese nor diabetic (Gottwald et al., 2010; Nevadunsky et al., 2014). This may be explained our hypothesis that higher educational attainment reduces the risk of EC through the pathway of reducing the risk factors of EC, such as obese and type 2 diabetes.

Clarifying the relationship between educational attainment and EC can facilitate the understanding of the causes of EC and improve the prevention policy of EC. This is the first study to explore the causal association between educational attainment and EC. Although RCTs is the gold standard to measure causality, it is impractical to conduct it in this study for two main reasons. First, it is inevitable but unethical to limit the length of educational attainment to some of the participants. Second, educational attainment usually completed at the age of 30, while EC mainly happens at postmenopausal period, which means the time span between exposure (educational attainment) and outcome (EC) will be very long. MR approach is the closest approximation to RCTs at present, where SNPs were used to mimic the process of randomly allocating some participants to more educational attainment and other participants to less education, which provides an analogy to RCTs in a non-experimental (observational) setting.

### Strengths and limitations

Our study has important strengths. Our study derived robust the causal association between educational attainment and EC by integrating summary level data from 1,131,881 people. This sample size was sufficient to derive unbiased causal estimates. Besides, we used recent state of the art methodological developments to thoroughly explore the possibility of pleiotropy in our results. The consistent result of different sensitivity analyses indicated little evidence of pleiotropy. Finally, we used different SNPs associated with educational attainment from two different dataset of SSGAC to perform all Mendelian randomization analyses twice and obtained consistent results, which indicated the reliability and robustness of our causal estimates.

Our study also has some limitations. First, we notice that some possible overlap between the exposure GWAS participants and the outcome GWAS in Hunter Community Study (0.71%), Queensland Institute of Medical Research (1.51%), and UK Biobank (52.08%) as shown in Supplementary Table S1. Since the educational attainment GWAS data we obtained was summary level, we cannot derive one-to-one mapping of each SNP and see whether overlap exist. But we used another educational attainment datasets, where UK Biobank study was not included, to perform all the Mendelian randomization analyses and obtain consistent results. One possible reason is that vast majority of the participant overlap in the outcome GWAS data set occurred among the controls, only 0.52% occurred in EC cases. Besides, if the selected SNPs of educational attainment have great interleaving or overlapping with EC, or if most of the strong SNPs for educational attainment are strongly pleiotropic for EC, then the SNPs for EC may have pleiotropic effect on educational attainment as well. However, our reverse direction Mendelian randomization found a null estimate which indicated that our causal estimates were influenced little by potential overlap. Second, there might be some potential confounding in the SNPs of educational attainment in our study. As Lee et al. reported, the SNPs implicate genes involved in brain-development processes and neuron-to-neuron communication. The polygenic score derived from the results explains around 11%–13% of the variance in educational attainment and 7%–10% of the variance in cognitive performance. Under this scenario, the SNPs may influence EC through many other biological pathways related to brain or neuron. However, such scenario is less likely to lead to the consistent set of results we found across our sensitivity analyses. All of results from the different several sensitivity analyses were consistent to prove that little evidence of heterogeneity or pleiotropy in our causal estimates. In addition, despite gaps in our understanding of the biological mechanisms through which these 306 SNPs influence educational attainment, they are disproportionately found in genomic regions that regulate brain development. They are enriched for biological pathways involved in neural development, and they are preferentially expressed in neural tissue. As these 306 SNPs do not seem to have any expression or enrichment in uterus, this further narrows the scope for pleiotropy. Third, the SNPs in our study were derived from the populations of European ancestry. Therefore, the causal estimates in our study were not able to be generalized to other populations. Lastly, the effect of changing the genetic variants may not be the same as the effect of changing the modifiable exposure through other means. That means, changing the level of educational attainment by changing the number of alleles associated with high educational attainment may not be the same as increasing the level of educational attainment from a public health perspective. However, this hypothesis can only be verified when we have a sufficient understanding of the specific mechanism how genetic variants influence educational attainment. Therefore, when a reader tries to interpret the result of our study, it should be noted that the effect estimate does not necessarily map directly on to the potential effect of a clinical or public health intervention. That is because Mendelian randomization estimates a “lifetime” effect of the exposure. It can be interpreted that Mendelian randomization tells people something about “state,” but not about the specific interventions that alter those states. However, Mendelian randomization is a tool for us to understand the causal association between exposures and outcomes, and that understanding can help to direct where action is needed to effect change in outcome. Therefore, we still recommend paying attention to the improvement of educational attainment for the potential causal reduction risk for endometrial cancer.

### Conclusion

This study indicated that low educational attainment was a causal risk factor for EC, especially for EC with endometrioid histology. Low educational attainment might lead to EC through the mediator of obesity, high WHR, and diabetes.

## Data Availability

Publicly available datasets were analyzed in this study. This data can be found here: https://app.mrbase.org/.

## Abbreviations

BMI, body mass idex; CI, Confidence intervals; EC, Endometrial cancer; ECAC, Endometrial Cancer Association Consortium; EEC, Endometrioid endometrial carcinoma; E2C2, The Epidemiology of Endometrial Cancer Consortium; GIANT, Genetic Investigation of Anthropometric Traits; GWAS, Genome-wide association study; ILCCO, International Lung Cancer Consortium; IVs, Instrumental variables; IVW, Inverse-variance-weighted; MR, Mendelian randomization; MR-PRESSO, MR pleiotropy residual sum and outlier; NECs, Non-endometrioid endometrial carcinomas; OR, Odds ratio; RCTs, Randomized controlled trials; SD, Standard deviation; SNPs, Single nucleotide polymorphisms; SSGAC, Science Genetic Association Consortium; WHR, Waist-to-hip ratio.
