# Supplementary material for: Educational attainment and endometrial cancer: A Mendelian randomization study
Source: Front Genet. 2022 Nov 29;13:993731. doi: 10.3389/fgene.2022.993731 (PMC9744760; doi:10.3389/fgene.2022.993731)

Supplementary Figure 9 Scatter plot of 71 SNPs associated with educational attainment and their risk of endometrial cancer with non-endometrioid histology

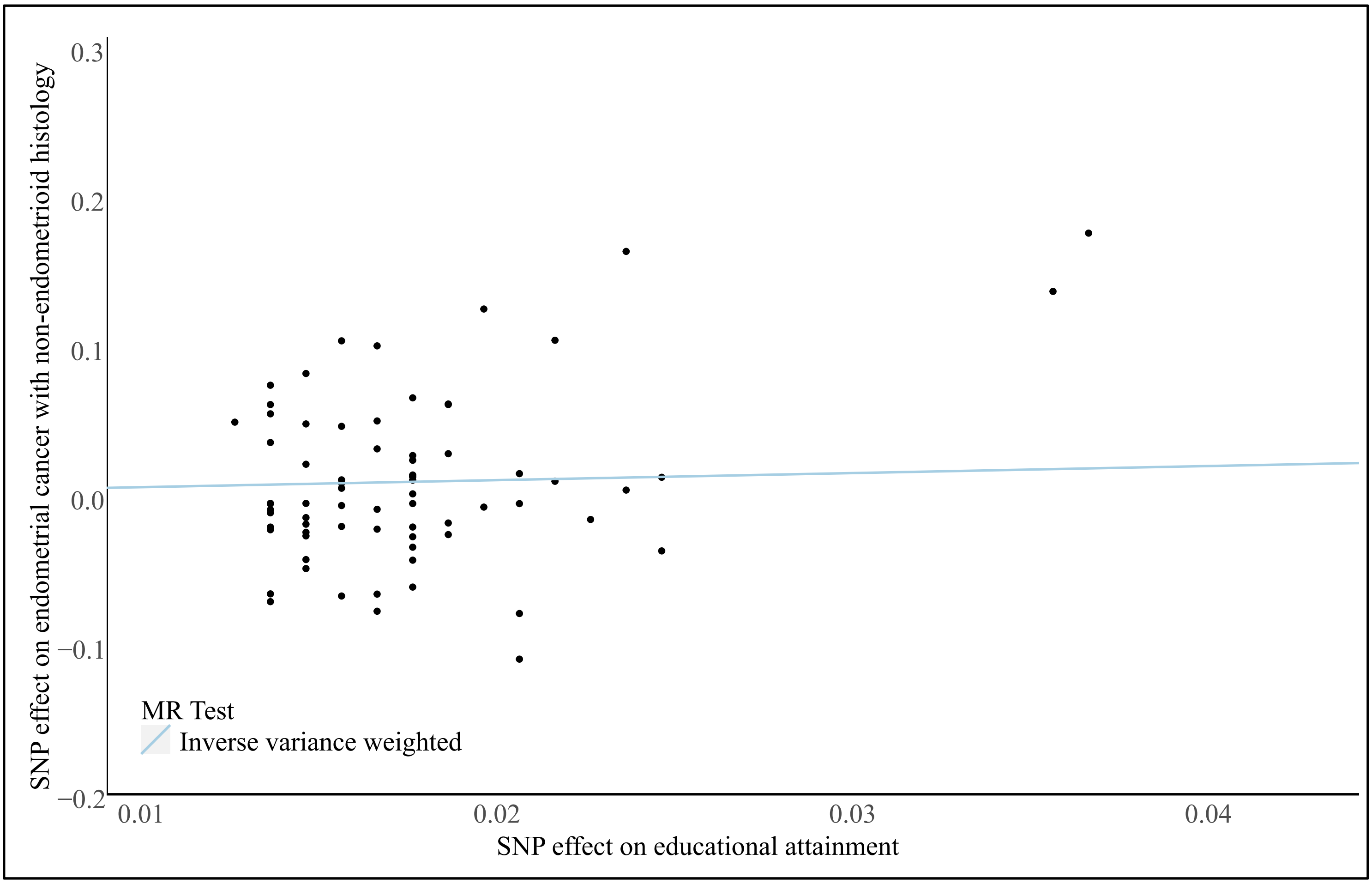

Supplement: Supplementary file 3 [file Image9.pdf]
