# Supplementary material for: Educational attainment and endometrial cancer: A Mendelian randomization study
Source: Front Genet. 2022 Nov 29;13:993731. doi: 10.3389/fgene.2022.993731 (PMC9744760; doi:10.3389/fgene.2022.993731)
Supplement: Supplementary file 5 [file Table7.docx]

Supplementary Table 7. Causal effect from endometrial cancer and educational attainment

| Method | Number of SNPs | OR | 95% CI | P-value |
| --- | --- | --- | --- | --- |
| Endometrial cancer |  |  |  |  |
| IVW method | 17 | 1.01 | 0.99-1.02 | 0.25 |
| Weighted-median method | 17 | 1.01 | 0.99-1.02 | 0.15 |
| MR Egger regression | 17 | 1.02 | 0.97-1.07 | 0.54 |
|  |  |  |  |  |
| Endometrial cancer with endometrioid histology |  |  |  |  |
| IVW method | 9 | 1.00 | 0.99-1.02 | 0.72 |
| Weighted-median method | 9 | 1.01 | 0.99-1.02 | 0.28 |
| MR Egger regression | 9 | 1.02 | 0.94-1.11 | 0.60 |

The sample size of Endometrial cancer with non-endometrioid histology was not enough for was to obtain SNPs of endometrial cancer with non-endometrioid histology. Therefore, the reverse mendelian randomization analysis on endometrial cancer with non-endometrioid histology was lacking. IVW, inverse variance weighted. OR, odds ratio. CI, confidence interval. SNPs, single nucleotide polymorphisms
