# Supplementary figures and images for: Educational attainment and endometrial cancer: A Mendelian randomization study
Source: Front Genet. 2022 Nov 29;13:993731. doi: 10.3389/fgene.2022.993731 (PMC9744760; doi:10.3389/fgene.2022.993731)

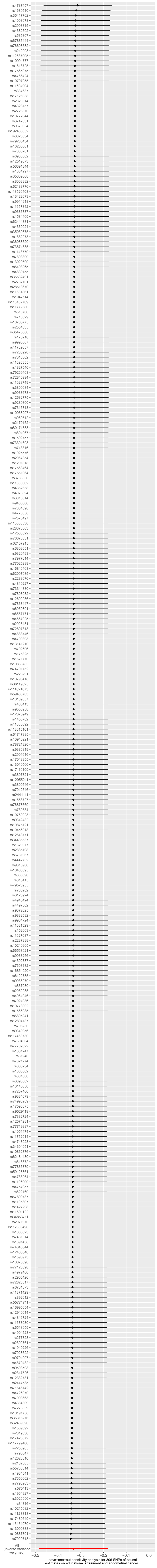

Supplement: Supplementary file 7 [file Image4.pdf]
