# Supplementary material for: Educational attainment and endometrial cancer: A Mendelian randomization study
Source: Front Genet. 2022 Nov 29;13:993731. doi: 10.3389/fgene.2022.993731 (PMC9744760; doi:10.3389/fgene.2022.993731)

Supplementary Figure 3   Funnel plot of 306 SNPS showing instrument strength against causal estimates

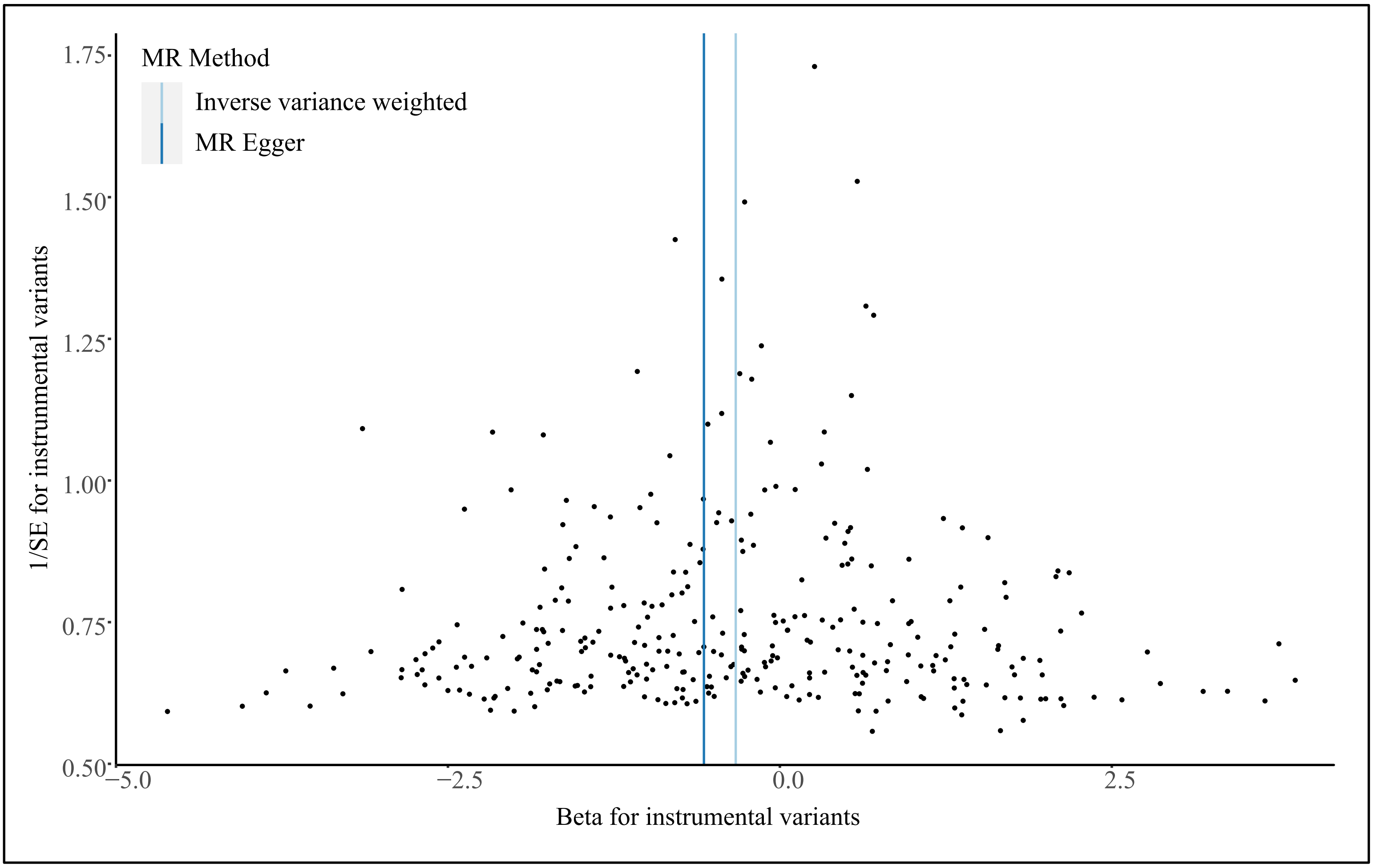

Supplement: Supplementary file 9 [file Image3.pdf]
