# Supplementary material for: Educational attainment and endometrial cancer: A Mendelian randomization study
Source: Front Genet. 2022 Nov 29;13:993731. doi: 10.3389/fgene.2022.993731 (PMC9744760; doi:10.3389/fgene.2022.993731)
Supplement: Supplementary file 10 [file Table5.docx]

Supplementary Table 5. Causal effect from educational attainment and endometrial cancer (SNPs obtained from SSGAC of 2016)

| Method | Number of SNPs | OR | 95% CI | P-value |  |
| --- | --- | --- | --- | --- | --- |
| Endometrial cancer |  |  |  |  |  |
| IVW method | 71 | 0.78 | 0.62-0.99 | 0.04 |  |
| Weighted-median method | 71 | 0.86 | 0.63-1.20 | 0.39 |  |
| MR-PRESSO test | 71 | 0.78 | 0.62-0.99 | 0.04 | Global test p = 0.252 |
| MR Egger regression | 71 | 0.80 | 0.23-2.81 | 0.74 |  |
|  |  |  |  |  |  |
| Endometrial cancer with endometrioid histology |  |  |  |  |  |
| IVW method | 71 | 0.68 | 0.51-0.90 | 0.008 |  |
| Weighted-median method | 71 | 0.63 | 0.42-0.94 | 0.02 |  |
| MR-PRESSO test | 71 | 0.68 | 0.51-0.90 | 0.009 | Global test p = 0.228 |
| MR Egger regression | 71 | 0.49 | 0.10-2.20 | 0.35 |  |
|  |  |  |  |  |  |
| Endometrial cancer with non-endometrioid histology |  |  |  |  |  |
| IVW method | 71 | 1.60 | 0.81-3.17 | 0.96 |  |
| Weighted-median method | 71 | 1.06 | 0.40-2.80 | 0.77 |  |
| MR-PRESSO test | 71 | 1.51 | 0.78-2.92 | 0.23 | Global test p = 0.316 |
| MR Egger regression | 71 | 29.36 | 0.92-932.78 | 0.13 |  |

IVW, inverse variance weighted. MR-PRESSO, MR pleiotropy residual sum and outlier. OR, dds ratio. CI, confidence interval.

SNPs, single nucleotide polymorphisms
