# Supplementary material for: Educational attainment and endometrial cancer: A Mendelian randomization study
Source: Front Genet. 2022 Nov 29;13:993731. doi: 10.3389/fgene.2022.993731 (PMC9744760; doi:10.3389/fgene.2022.993731)
Supplement: Supplementary file 16 [file Image1.pdf]

Supplementary Figure 1. Conceptual illustration of the MR method and its three underlying core assumptions

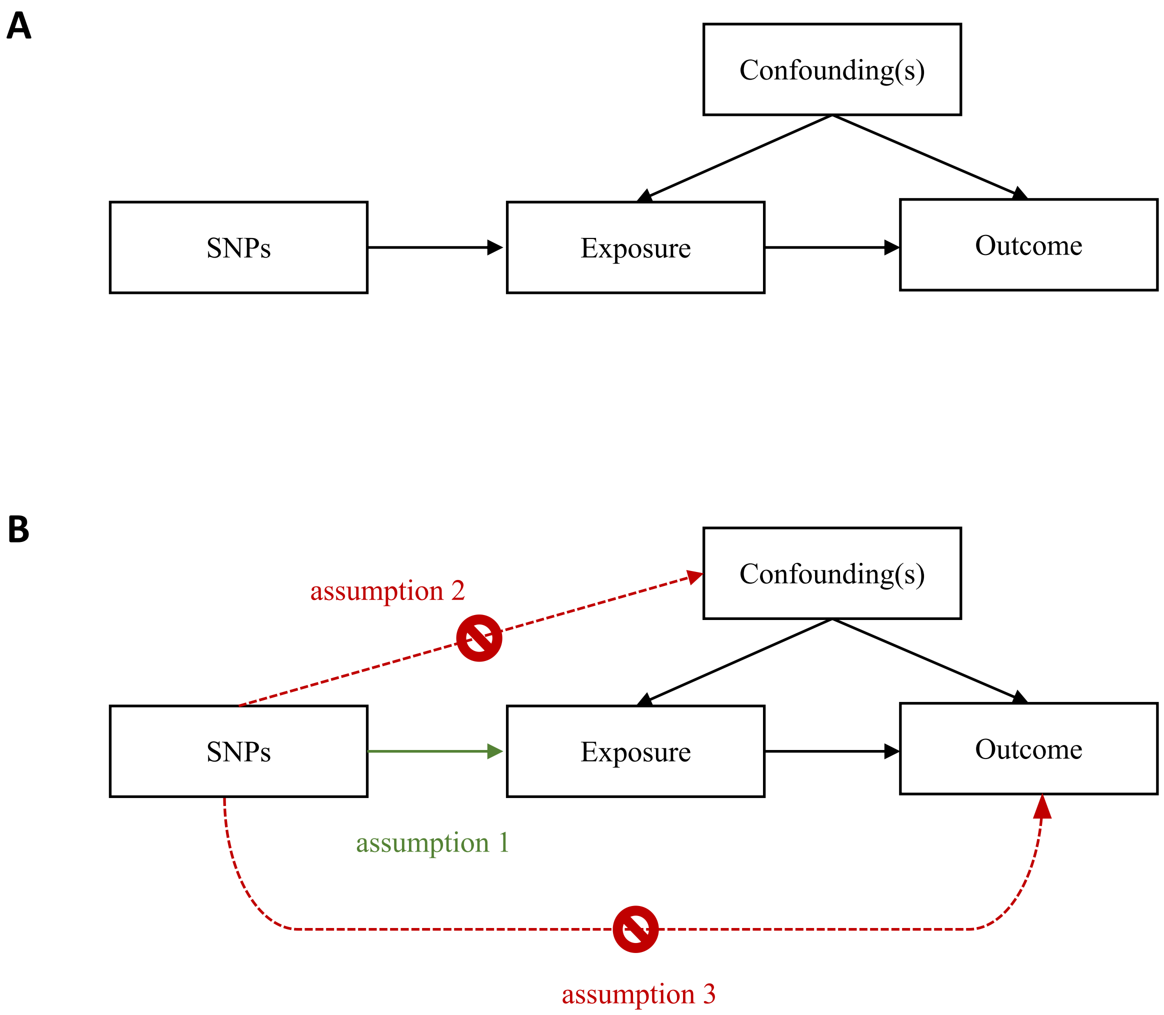

(A) Conceptual illustration of the MR method; (B) Three underlying core assumptions of MR method
